# Concentration Gradient-Induced Syntheses and Crystal Structures of Two Copper(II) Coordination Polymer Based on Phthalic Acid and 2,2′-Bipyridine

**DOI:** 10.3390/molecules30091953

**Published:** 2025-04-28

**Authors:** Tao Zhou, Gengyi Zhang, Chunhong Tan, Yong Liu, Xiao-Feng Wang

**Affiliations:** 1 School of Chemistry and Chemical Engineering, University of South China, Hengyang 421001, China; 15774023961@163.com; 2 School of Resources Environment and Safety Engineering, University of South China, Hengyang 421001, China; leoleoleo111@126.com

**Keywords:** multi-copper cluster, coordination polymer, gradient-induced, crystal structure

## Abstract

The reaction of copper nitrate, phthalic acid (1,2-H_2_BDC), and bipyridine in ammonia/ethanol media affords two multi-copper (II) cluster-based coordination polymers, namely {[Cu_4_(bpy)_4_(OH)_2_(BDC)_2_]·2OH·13H_2_O}_n_ (**USC-CP-6**) and {[Cu_2_(BDC)_2_(bpy)_2_(H_2_O)]·3H_2_O}_n_ (**USC-CP-7**), under ambient conditions, with **CP-6** forming at the bottom and **CP-7** at the upper edge of the same beaker. The single-crystal structures reveal that it is a rare case of gradient-induced formation of different multi-copper(II) cluster-based CPs within a single-solution chemical reaction. **CP-6** crystallizes in the monoclinic system, sp. gr. *P*2_1_/c, and is composed of chair-like tetranuclear [Cu_4_(*μ*_3_-OH)_2_(bpy)_4_(BDC)_2_]^2+^ clusters as secondary building units, bridged by BDC^2−^ ligands to form a two-dimensional layer framework, while **CP-7** crystallizes in the monoclinic system, sp. gr. *P*2_1_/n, with binuclear [Cu_2_(1,2-BDC)_2_(bpy)_2_(H_2_O)] clusters linked by bridging BDC^2−^ ligands to form a one-dimensional looped double chain. Through intermolecular *π*–*π* stacking and hydrogen bonds between the coordination water, lattice water, and free oxygen atoms from carboxylate, both compounds yield a 3D supramolecular structure.

## 1. Introduction

Over the past two decades, coordination polymers (CPs) have been extensively studied as a kind of promising material with potential applications in the areas of chemical sensors, magnetism, adsorption, and catalysis [1,2,3,4,5,6,7]. Their consequentially rational design has rapidly expanded due to the versatile structural motifs and intriguing varieties of architectures. In principle, the desired structure can be obtained by selecting appropriate organic ligands and designed metal clusters. Specifically, the structural control of multinuclear metal cluster-based CPs can be achieved by choosing suitable organic linkers combined with d- and f-block metal clusters as secondary building units (SBUs) under optimized reaction conditions [8,9,10,11]. However, the inherent coordination flexibility of metal ions and organic ligands leads to a degree of structural unpredictability. Thus, these CPs are typically synthesized via a one-pot approach rather than through the theoretically possible but practically challenging stepwise methodology involving the pre-assembly of discrete polynuclear metal clusters followed by ligand incorporation [12,13,14]. Therefore, the development of such new materials is challenging, as it is still difficult to accurately control and predict their structures in the assembly process, especially mixed-ligand-based multinuclear metal complexes [15].

The exploration generally relies only on measurements of the structures of isolated product states, namely, the crystal. Despite the development of spectroscopic techniques, X-ray diffraction crystallographic analysis remains the best source of information on the structure of chemical compounds, including CPs. It is a powerful scientific tool for determining the molecular structure of crystals and has been extended to studying different types of intermolecular interactions for a deeper understanding of the crystal growth process and various experimental parameters that influence their structures [16,17].

Many reaction parameters can play an important role in affecting the construction of coordination frameworks, such as the reaction temperature, pH value, solvent, template agents, counter-ions, and concentration of reactants [18,19,20]. A subtle alteration in these factors can lead to a drastic change in the structure in terms of SBU, dimension, and topology [21,22]. CPs can be quickly obtained (in half a day to a few days) through hydro-/solvothermal reaction in an autoclave, but it is a seemingly black-box-like process, and it is quite difficult to control the designed SBU prototype [23,24]. In contrast, solution chemical reaction in an open system, which is a very slow approach (a few weeks or months) and may randomly suspend the reaction process to isolate the intermediate product, provides substantially better opportunities to deeply understand the assembly formation mechanism of CPs and can yield variable polynuclear metallic cluster-based coordination compounds [25,26,27].

Under open-solution chemical reaction systems, we directly observed the layer-by-layer growth process of a three-dimensional coordination polymer (CP), the controllable self-assembly of kinetically and thermodynamically stable multinuclear copper complexes, and the entire pathway from discrete cluster-based hydrogen networks to layer metal-carboxylate coordination polymers [28,29,30].

In our previous work, we explored the synthesis of copper complexes using multi-carboxylic acids and N-donor co-ligands. However, using benzene tricarboxylate (H_3_BTC), we reported a copper complex based on rare asymmetric chair-like tetranuclear [Cu_4_(*μ*_2_-OH)_2_(*μ*_3_-OH)_2_(H_2_O)(BTC)] clusters [31], rather than chair-like-type tetranuclear [Cu_4_(*μ*_2_-OH)_2_(*μ*_3_-OH)_2_(X)_2_] (X = H_2_O, Cl, etc.) cationic cores [32,33]. In the further case of phthalic acid replacing H_3_BTC, it is interesting to note that we found the formation of two different copper coordination complexes induced by the concentration gradient of the same beaker, which are chain and layer structures based on variable SBUs.

Herein, the reaction of copper nitrite, phthalic acid (1,2-H_2_BDC), N-donor cheating co-ligand 2,2′-bipyridine (bipy) in ammonia solution yielded two multi-copper(II) cluster-based coordination compounds, {[Cu_4_(bpy)_4_(OH)_2_(BDC)_2_]·2OH·13H_2_O}_n_ (**USC-CP-6**) and {[Cu_2_(BDC)_2_(bpy)_2_(H_2_O)]·3H_2_O}_n_ (**USC-CP-7**, where USC-CP stands for University of South China coordination polymer), whose structures indicated the formation of a layered metal-carboxylate coordination framework and a looped coordination compound induced by the concentration gradient of the same reaction system.

## 2. Results

### 2.1. Synthesis

**Scheme 1:**
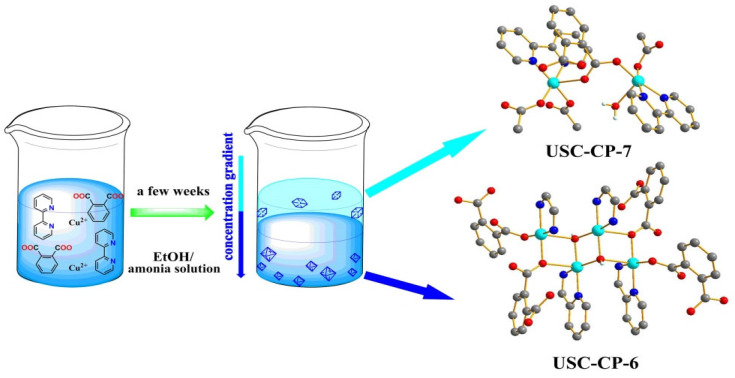
The self-assembly preparation of two CPs induced by concentration gradient.

Polynuclear copper(II) clusters are of special interest not only due to the central role they play in biological systems but also due to interest in the establishment of magneto-structural correlations [34,35]. A variety of hydroxo-bridged dinuclear copper(II) complexes with [Cu(*μ*_2_-OH)_2_Cu] cores have been reported, and they can be assembled to generate chair-like-type tetranuclear [Cu_4_(*μ_2_*-OH)_2_(*μ_3_*-OH)_2_] cationic cores [32]. When multi-carboxylic acids with N-donor co-ligands were employed, tetranuclear copper complexes were prepared, in which the chair-like-type hydroxy-bridged Cu_4_ cores are often symmetric clusters [36,37]. When using benzene tricarboxylate (H_3_BTC), a rare asymmetric chair-like-type tetranuclear copper complex was reported in our previous work [31]. In fact, as the placement and reaction progressed, a concentration gradient was established in the reaction beaker from top to bottom. Theoretically, this gradient should have an impact on the chemical reaction. However, irrespective of the evolution of circumstances, to date, only a single product has been separated and reported within a single-solution reaction system. Through strategic ligand substitution of H_3_BTC with phthalic acid (1,2-H_2_BDC) [31], we aimed to modulate the structures of hydroxy-bridged Cu_4_ cores, and the formation of block blue crystals (**USC-CP-6**) was observed at the base of the beaker after two weeks, while the color of the mixture solution faded to a lighter shade. We adopted a routine procedure to pick up fine crystals in order to analyze the crystal structure. Through meticulous single-crystal X-ray diffraction analysis (requiring multiple measurements to resolve lattice water molecules in **USC-CP-6**), we serendipitously picked up a single crystal and characterized a coexisting dinuclear phase (**USC-CP-7**) from upper solution regions lacking hydroxyl bridges. During the longer experimental procedure, the color of the mixture solution appeared in an obvious gradient, with the upper part turning light blue, and single crystals of **USC-CP-7** were relatively easy to select at the upper edge of the beaker (Scheme 1). These results demonstrate that, to a certain degree, the occurrence of a solution chemical reaction may result in a mixture of products in which one of these products accounts for the vast majority, and thereby even PXRD does not reveal any impurity phases. Our study is a rare case in which this reactant concentration gradient plays an important role in inducing variable crystal structures in the solution chemical reaction system.

### 2.2. Molecular Structure and Hydrogen Bonds of **USC-CP-6**

**Figure 1:**
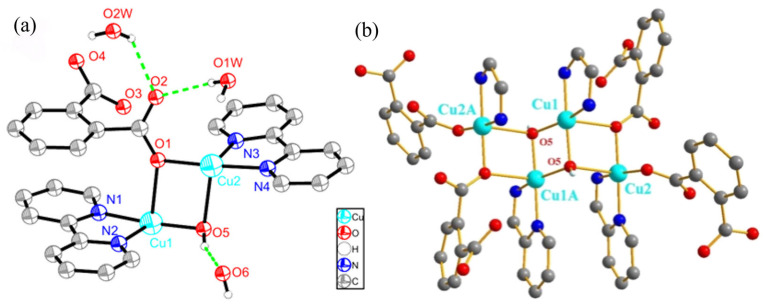
ORTEP view of the asymmetric unit in **CP-6** with displacement ellipsoids (30%) and atomic labeling (**a**) and the tetranuclear cation [Cu_4_(bpy)_4_(OH)_2_(COO)_4_]^2+^ (**b**). The green dotted lines represent hydrogen bonds.

The X-ray single-crystal structure reveals that **CP-6** crystallizes in a monoclinic system with the space group *P*2_1_/c (Table 1). The asymmetric unit contains two independent Cu(II) ions, two bpy, one BDC, and a hydroxide anion, and 13 lattice H_2_O molecules, and it can be expanded as a chair-like symmetric [Cu_4_(*μ*_3_-OH)_2_(bpy)_4_(1,2-BDC)_2_]^2+^ cluster as a secondary building unit (see Figure 1). Each center Cu(II) ion (Cu1 and Cu1A) is coordinated by two N atoms of bpy and two O atoms from different *μ*_3_-OH groups in the equatorial plane, while one O atom from the mono-dentate bridging O atom from carboxylate group of 1,2-BDC^2−^ occupies the axial position. *π*–*π* interactions are observed between the pyridine rings, with a center-to-center distance of 3.633(1) Å (Figure S1). The distances of Cu-N are about 2 Å, while the range of the Cu–O bond lengths is 1.950(3)–2.393(3) Å (Table S1). The equatorial plane of both terminal Cu(II) ions (Cu2 and Cu2A) is coordinated by two nitrogen atoms of bpy, one oxygen atom of the bridging carboxylate O atom from one 1,2-BDC ligand, and one O atom of the monodentate terminal carboxylate from another 1,2-BDC ligand (the Cu–O bond lengths are 1.963(3) and 2.368(3) Å for Cu2 and 1.974(3) and 2.393(3) Å for Cu2A, respectively), while the apical position is occupied by an O atom of *μ*_3_-OH with a Cu2-O1 length of 1.974(3) Å. The two crystallographically independent Cu(II) ions (Cu1 and Cu2) both exhibit a distorted trigonal bipyramidal geometry, as evidenced by their *τ*_5_ values of 0.28 and 0.10, respectively. The smaller *τ*_5_ value for Cu2 indicates a geometry closer to a square pyramid (*τ*_5_ = 0 for an ideal square pyramid; *τ*_5_ = 1 for an ideal trigonal bipyramid) [38,39]. The structural parameters of **CP-6**, including bond lengths, angles, and coordination geometries, fall within the expected ranges for complexes of this type [40,41,42,43,44,45].

**Scheme 2:**
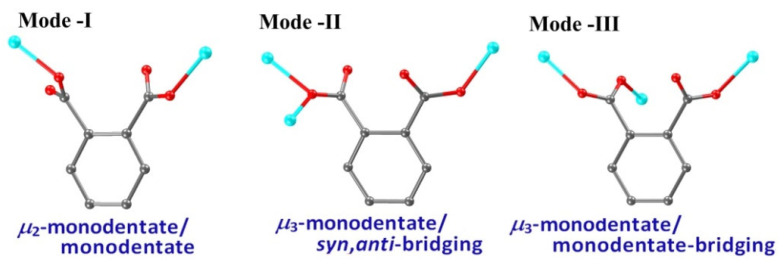
Coordination modes of the dicarboxylic acid ligand. Grey represents carbon, blue represents cobalt, and red represents oxygen.

**Figure 2:**
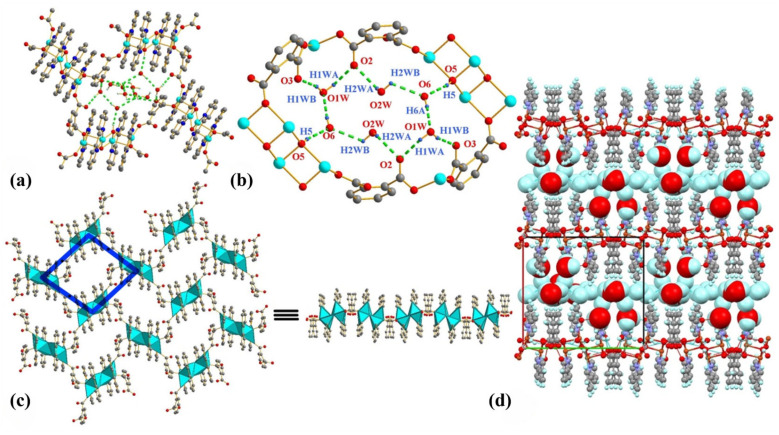
The H-bonds (green dotted line) between four adjacent Cu_4_ clusters (**a**) and the details (**b**); vertical and side views of the single chain (**c**); and packing along the *c*-axis with the space-filled water molecules between adjacent layers (**d**) in **CP-6**. The red and white spheres represent water molecules.

The fully deprotonated 1,2-BDC^2−^ acts as a bidentate ligand bridging three Cu(II) atoms through two O atoms from two carboxylates: a ligated one to a Cu(II) atom, and another bridging two Cu(II) atoms (Mode I in Scheme 2). Every 1,2-BDC^2−^ ligand links four adjacent [Cu_4_(bpy)_4_(OH)_2_(1,2-BDC)_2_]^2+^ SBUs to form an infinite two-dimensional 4 × 4 framework with argyle apertures, which are filled with lattice water molecules. Naturally, the structure optimizes the hydrogen bonding of the O-H···O type. As depicted in Figure 2b, all hydroxyl, water, and carboxylate groups participate in the formation of sixteen-membered ring-like hydrogen bonds. The lattice waters and two coordinated *μ*_2_-OH groups serve as hydrogen bond donors. The uncoordinated O atoms from the monodentate terminal carboxylate of 1,2-BDC act as hydrogen bond acceptors. Additionally, the two uncoordinated hydroxyl groups (OH^−^) function both as hydrogen bond donors and acceptors, thereby forming three distinct types of hydrogen bonds. Furthermore, the layers slide-pack to form a 3D supramolecular structure through the extensive hydrogen bonds of lattice water molecules (see Table 2 and Figure 2).

### 2.3. Molecular Structure and Hydrogen Bonds of **USC-CP-7**

**Figure 3:**
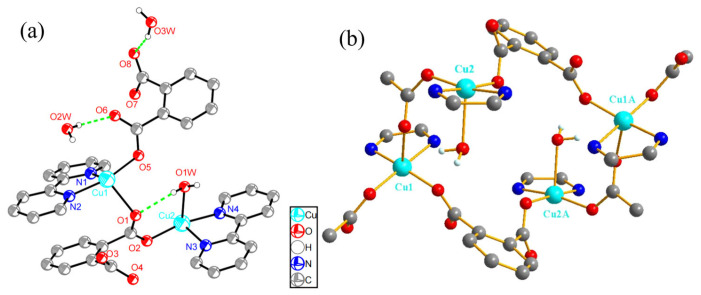
ORTEP view of the asymmetric unit in **CP-7** with displacement ellipsoids (30%) (**a**) and atomic labeling, and the tetranuclear loop [Cu_4_(bpy)_4_(BDC)_2_(COO)_4_] (**b**). The green dotted lines represent hydrogen bonds.

Single-crystal X-ray diffraction analysis reveals that **CP-7** crystallizes in the monoclinic system with the space group *P*2_1_/n (Table 1). The asymmetric unit contains two crystallographically unique copper ions, two fully deprotonated 1,2-BDC^2−^ ligands, two bpy, one coordinated H_2_O molecule, and three lattice water molecules, and it can be expanded as a chair-like symmetric [Cu_4_(bpy)_4_(1,2-BDC)_2_(H_2_O)(COO)_4_] cluster as a secondary building unit. As depicted in Figure 3, both Cu^2+^ ions are five-connected; Cu1 is coordinated by two N atoms of chelating bpy and two O atoms of monodentate carboxylate groups from different 1,2-BDC^2−^ ligands in the equatorial plane (with Cu1-O3 = 1.941(3) and Cu1-O5 = 1.956(2) Å), while one O atom from bidentate bridging carboxylate group of 1,2-BDC^2−^ occupies the axial position, with a Cu1-O1 length of 2.368(3) Å. The equatorial plane of Cu2 is also coordinated by two nitrogen atoms of bpy, two oxygen atoms from mono-dentate and bridging carboxylates from two 1,2-BDC^2−^ ligands (with Cu2-O2 = 1.972(2), Cu2-O7 = 1.963(3)Å), while the apical position is occupied by the O atom of a coordinated H_2_O molecule with a Cu2-O1W length of 2.312(3) Å. These observed bond lengths and angles show excellent agreement with previously reported values for analogous complexes [40,41,42,43,44,45]. The two crystallographically independent Cu(II) ions (Cu1 and Cu2) both exhibit a distorted trigonal bipyramidal geometry, as evidenced by their *τ*_5_ values of 0.16 and 0.07, respectively. The smaller *τ*_5_ value for Cu2 indicates a geometry closer to a square pyramid [38,39]. There are two kinds of fully deprotonated 1,2-BDC^2−^ ligands adopting different coordination fashions. One acts as a *μ*_3_-bridging ligand to ligate three Cu(II) ions through two types of carboxylate groups: (i) monodentate and (ii) bidentate in syn-anti bridging (modes II and III in Scheme 2). The other acts as a *μ*_2_-bridging ligand to ligate two Cu(II) ions through two monodentate carboxylates. The tetranuclear secondary building units are bridged by *μ*_2_-BDC in order to construct a one-dimensional infinite looped double chain (Figure 3), which contains *π*–*π* stacking between *μ*_3_-BDC and bpy with a distance of 3.610(9) Å (see Figure S2).

**Figure 4:**
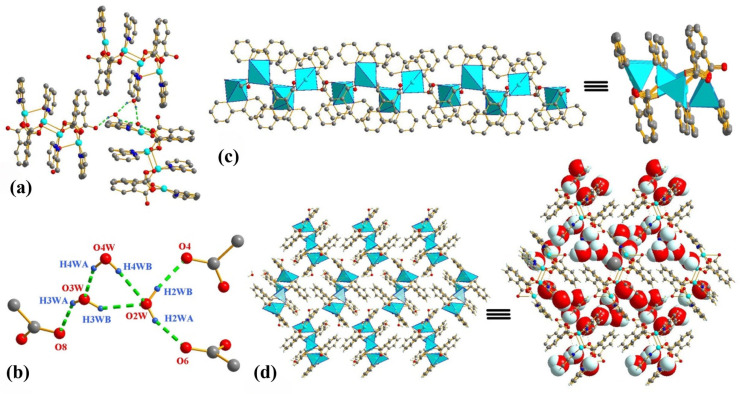
The H-bonds (green dotted line) between three adjacent chains (**a**) and the details (**b**); vertical and side views of the single chain (**c**); and packing along the *c*-axis (**d**) in **CP-7**. The red and white spheres represent water molecules.

Inside the adjacent three chains, there is a triangular six-membered H-bond ring among three lattice water molecules (O2W, O3W, and O4W), which further form H-bonds with three O atoms from carboxylate groups. As illustrated in Figure S3, through the *π*–*π* stacking of *μ*_2_-BDC from adjacent chains (the center-to-center distance is 4.000(1) Å), the 1D looped chains construct the wave-like supramolecular layer (Figure S3). As shown in Figure 4b, lattice water molecules and carboxylate groups form triangle-like hydrogen bonds. Specifically, lattice water molecules act as hydrogen bond donors, while the uncoordinated O atoms from the monodentate terminal carboxylate of 1,2-BDC serve as hydrogen bond acceptors. This interaction results in three distinct types of hydrogen bonds: intermolecular hydrogen bonds between coordinated water and lattice water, lattice water and lattice water, and lattice water and free oxygen atoms from monodentate carboxylate (Table 3). Furthermore, the wave-like layers of **CP-7** are stabilized by the presence of numerous hydrogen bonds, which are extended to yield a 3D supramolecular framework (Figure 4).

## 3. Materials and Methods

### 3.1. Synthesis of **CP-6** and **CP-7**

A solution of 2,2′-bipyridine (0.156 g, 0.6 mmol) in EtOH (5 mL) was added dropwise to a stirred solution of Cu(NO_3_)_2_·3H_2_O (0.145 g, 0.6 mmol) and 1,2-benzenedicarboxylic acid (0.066 g, 0.4 mmol) in 20 mL of ammonia solution (28% in water), and then the mixture was stirred continuously for 30 min to yield a dark blue solution. The dark blue filtrate was allowed to stand at room temperature for several weeks to give deep-blue block crystals of **CP-6** at the bottom and **CP-7** at the upper edge of the beaker, respectively. Yield: 13% and 5% for **CP-6** and **CP-7,** respectively (based on Cu). Elemental analysis calculated for {[Cu_4_(bpy)_4_(OH)_2_(BDC)_2_]·2OH·13H_2_O}_n_ (**CP-6**): C 13.42, H 3.59, N 10.58; found: C 13.22, H 4.72, N 10.69%. For {[Cu_2_(BDC)_2_(bpy)_2_(H_2_O)]·3H_2_O}_n_ (**CP-7**): C 19.87, H 2.48, N 11.59; found: C 19.42, H 2.99, N 11.38%.

### 3.2. Crystallographic Studies

All data were collected on a Rigaku MM007-HF CCD (Saturn724+, Rigaku Corporation, Tokyo, Japan) diffractometer with graphite-monochromated Mo-Kα radiation (λ = 0.71073 Å) at 293 K. Data processing, including empirical absorption corrections, was performed by the multi-scan technique using the Crystal Clear 1.3.6 software package (Rigaku/MSC, Tokyo, Japan, 2005). The structure was solved by direct methods and refined by the full-matrix least-squares method on *F*^2^ with the SHELXTL program package [46]. All non-hydrogen atoms of the frameworks were refined with anisotropic displacement parameters. The hydrogen atoms of organic ligands were placed in geometrically calculated positions and refined using the riding model, while the hydrogen atoms bonded to oxygen were located in a difference Fourier map. The crystallographic data have been deposited into the Cambridge Crystallographic Data Centre, CCDC nos. 2435678 and 1988874 for **USC-CP-6** and **USC-CP-7**, respectively. Copies of this information may be obtained free of charge from the Director, 12 Union Road, Cambridge CB2 1EZ, UK; fax: +44-1223-336033; e-mail: deposit@ccdc.cam.ac.uk. Crystal data collection and refinement parameters are given in Table 1. Selected bond lengths and angles are listed in Tables S1 and S2 in the Supplementary Material, respectively.

## 4. Conclusions

In summary, we successfully synthesized and isolated two multinuclear copper coordination polymers based on Cu_4_ cluster secondary building units. The structures of the two copper CPs were determined by single-crystal X-ray diffraction analysis. In **CP-6**, the hydroxy-bridged tetra-copper(II) cluster, Cu_4_(bpy)_4_(OH)_2_(1,2-BDC)_2_, as a secondary building unit, is bridged by four 1,2-BDC^2−^ ligands to form a 2D layer, which is further packed by extensive hydrogen bonds of lattice water molecules to yield a 3D supramolecular framework. In **CP-7**, four Cu(II) ions are linked by two bridging 1,2-BDC^2−^ ligands to form a Cu_4_(μ_3_-BDC)_2_ loop without coordinated hydroxyl groups. This loop acts as a secondary building unit and is subsequently bridged by *μ*_2_-BDC to generate a one-dimensional infinite double-chain structure with Cu_2_(*μ*_2_-BDC)_2_ loops. These chains are further packed through sliding interactions mediated by extensive hydrogen bonding among lattice water molecules, resulting in a 3D supramolecular framework. Notably, both compounds were obtained from the same reaction system (ammonia/ethanol solution containing bpy and benzene dicarboxylic acid) through a concentration gradient approach—a rare phenomenon that yields distinct multinuclear cluster-based CPs from identical starting materials.

## Figures and Tables

**Table 1:** Crystallographic characteristics and X-ray data collection and structure-refinement parameters for **CP-6** and **CP-7**.

Parameter	CP-6	CP-7
Formula	C_56_H_70_Cu_4_N_8_O_25_	C_36_H_32_Cu_2_N_4_O_12_
Mr	1509.36	839.73
Temp (K)	296	293
Cryst system	monoclinic	monoclinic
Space group	*P*2_1_/c	*P*2_1_/n
*a*/Å	16.609(5)	12.992(5)
*b*/Å	16.984(5)	20.709(7)
*c*/Å	13.707(4)	13.652(5)
*β*/°	110.014(5)	109.401(7)
*V*/Å^3^	3633.0(18)	3465(2)
*Z*	2	4
*D*_c_/g cm^−3^	1.380	1.610
*μ*/mm^−1^	1.232	1.300
*F* (000)	1556	1720
*R* (int)	0.0395	0.0743
Total reflections	36,757	28,530
Unique reflections	7184	4353
*I* > 2σ (*I*)	8311	6101
*R*_1_	0.0862	0.0624
*wR*_2_	0.2622	0.1123
*S*	1.155	0.929

**Table 2:** Hydrogen bond geometry for **CP-6**.

D-H···A	D-H, Å	H···A, Å	D···A, Å	D-H···A, deg
O5-H5···O6	0.85	2.04	2.866(4)	158
O6-H6···O1W	0.85	2.11	2.854(5)	143
O1W-H1WA···O2	0.85	2.19	2.803(6)	145
O1W-H1WB···O3	0.85	1.86	2.694(4)	166
O2W-H2WA···O2	0.85	2.05	2.878(4)	128
O2W-H2WB···O6	0.85	2.10	2.858(6)	132

**Table 3:** Hydrogen bond geometry for **CP-7**.

D-H···A	D-H, Å	H···A, Å	D···A, Å	D-H···A, deg
O1W-H1WA···O1	0.85	2.16	2.880(4)	142
O1W-H1WB···O2W	0.85	2.03	2.870(5)	168
O2W-H2WA···O6	0.85	2.25	3.088(5)	170
O2W-H2WB···O4	0.85	2.10	2.886(6)	154
O3W-H3WB···O2W	0.85	2.11	2.875(5)	149
O3W-H3WA···O8	0.85	2.15	2.806(5)	134
O4W-H4WB···O3W	0.85	1.96	2.761(4)	157
O4W-H4WB···O2W	0.85	1.91	2.753(4)	172

## Data Availability

The original contributions presented in this study are included in the article/Supplementary Material. Further inquiries can be directed to the corresponding authors.

## Supplementary Materials

The following supporting information can be downloaded at: https://www.mdpi.com/article/10.3390/molecules30091953/s1. Table S1. Selected bond distances d (Å) and bond angles ω (deg) for the **CP-6**. Table S2. Selected bond distances d (Å) and bond angles ω (deg) for the **CP-7**. Figure S1. The *π*–*π* stacking between bpy of the Cu_4_-cluster of complex **CP-6**. All hydrogen and some carbon atoms of bpy are omitted for clarity. Figure S2. BDC bridging adjacent Cu_4_(*μ*_3_-BDC)_2_-loop to form the chain (left), and the *π*–*π* stacking between *μ*_2_-BDC and bpy of **CP-6** (right). All hydrogen and some carbon atoms of bpy are omitted for clarity. Figure S3. The *π*–*π* stacking of *μ*_2_-BDC from adjacent chains (left), and packing diagram of **CP-7** along the *c*-axis (right). Hydrogen atoms bonded to C atoms are omitted for clarity.
