# Supplementary material for: Concentration Gradient-Induced Syntheses and Crystal Structures of Two Copper(II) Coordination Polymer Based on Phthalic Acid and 2,2′-Bipyridine
Source: Molecules. 2025 Apr 28;30(9):1953. doi: 10.3390/molecules30091953 (PMC12074063; doi:10.3390/molecules30091953)
Supplement: Supplementary file 1 [file molecules-30-01953-s001.zip › molecules-3605487-supplementary.pdf]

SUPPORTING INFORMATION

Concentration Gradient Induced Syntheses and Crystal Structures of Two Copper(II) Coordination Polymer Based on Phthalic Acid and 2,2′-bipyridine

Tao Zhou[1,2], Gengyi Zhang[2], Chunhong Tan[1,*], Yong Liu[2,*], Xiao-Feng Wang[1,*]

[1]*School of Chemistry and Chemical Engineering, University of South China, Hengyang, 421001, P. R. China.*

[2]*School of Resources Environment and Safety Engineering, University of South China, Hengyang, 421001, P. R. China.*

Table S1. Selected bond distances d (Å) and bond angles ω (deg) for the **CP-6**

Bond	d	Bond	d
Cu1-O5	1.950(3)	Cu2-O4	1.948(3)
Cu1-O5	1.963(3)	Cu2-O1	1.974(3)
Cu2-N2	2.003(4)	Cu2-N4	1.998(4)
Cu1-N1	1.993(4)	Cu2-N3	2.015(4)
Cu1-O1	2.406(3)	Cu2-O5	2.393(3)
Angle	ω	Angle	ω
O5-Cu1-O5	78.96(13)	O4-Cu2-O1	87.77(13)
O5-Cu1-N1	175.18(14)	O4-Cu2-N4	96.50(15)
O5-Cu1-N1	99.57(14)	O1-Cu2-N4	175.38(14)
O5-Cu1-N2	101.73(14)	O4-Cu2-N3	169.68(14)
O5-Cu1-N2	158.35(14)	O1-Cu2-N3	94.46(14)
N1-Cu1-N2	81.34(16)	N4-Cu2-N3	81.03(16)
O5-Cu1-O1	81.45(11)	O4-Cu2-O5	93.67(12)
O5-Cu1-O1	108.31(12)	O1-Cu2-O5	81.32(11)
N1-Cu1-O1	94.75(13)	N4-Cu2-O5	100.12(13)
N2-Cu1-O1	93.10(13)	N3-Cu2-O5	96.63(12)

Table S2. Selected bond distances d (Å) and bond angles ω (deg) for the **CP-7**

Bond	d	Bond	d
Cu1-O5	1.941(3)	Cu2-O7	1.963(3)

Cu1-O5	1.956(2)	Cu2-O2	1.972(2)
Cu1-N2	2.013(3)	Cu2-N4	2.022(3)
Cu1-N1	2.010(3)	Cu2-N3	2.032(3)
Cu1-O1	2.368(3)	Cu2-O1W	2.312(3)
Angle	ω	Angle	ω
O3-Cu1-O5	88.04(12)	O7-Cu2-O2	89.69(11)
O3-Cu1-N1	176.91(12)	O7-Cu2-N4	94.11(11)
O5-Cu1-N1	93.05(12)	O2-Cu2-N4	168.22(11)
O3-Cu1-N2	97.52(12)	O7-Cu2-N3	172.57(12)
O5-Cu1-N2	167.01(11)	O2-Cu2-N3	94.78(12)
N2-Cu1-O1	106.48(10)	N1-Cu1-N2	80.82(12)
N1-Cu1-O1	91.70(11)	N4-Cu2-O1W	89.17(11)
O3-Cu1-O1	91.27(10)	N4-Cu2-N3	80.37(12)
O5-Cu1-O1	85.03(10)	O7-Cu2-O1W	97.22(11)
		O2-Cu2-O1W	101.42(10)
		N3-Cu2-O1W	87.72(12)

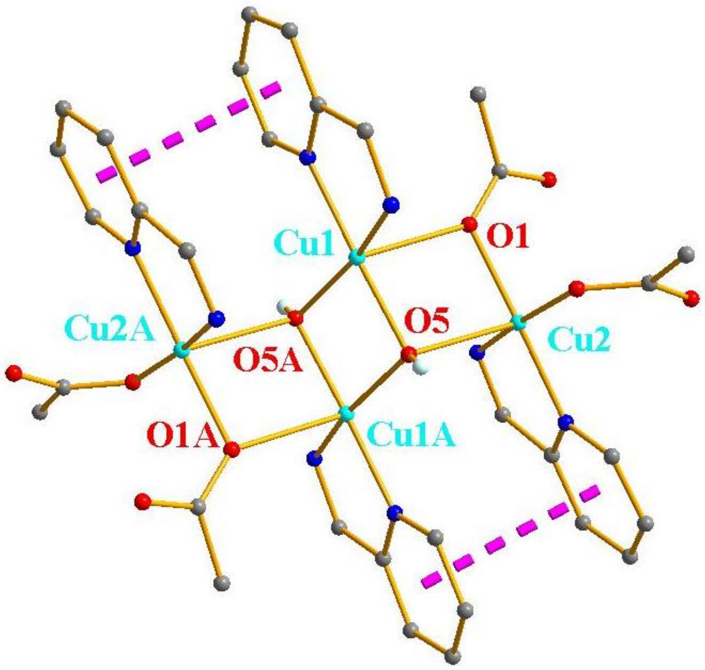

Figure S1. The π-π stacking between bpy of the Cu_4-cluster of complex **CP-6**. All hydrogen and some carbon atoms of bpy are omitted for clarity.

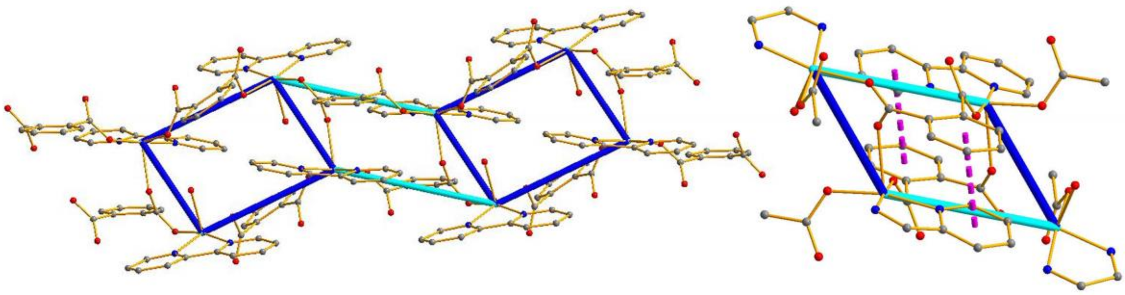

Figure S2. BDC bridging adjacent $Cu_4(\mu_3\text{-BDC})_2$-loop to form the chain (left), and the π-π stacking between μ_2-BDC and bpy of **CP-6** (right). All hydrogen and some carbon atoms of bpy are omitted for clarity.

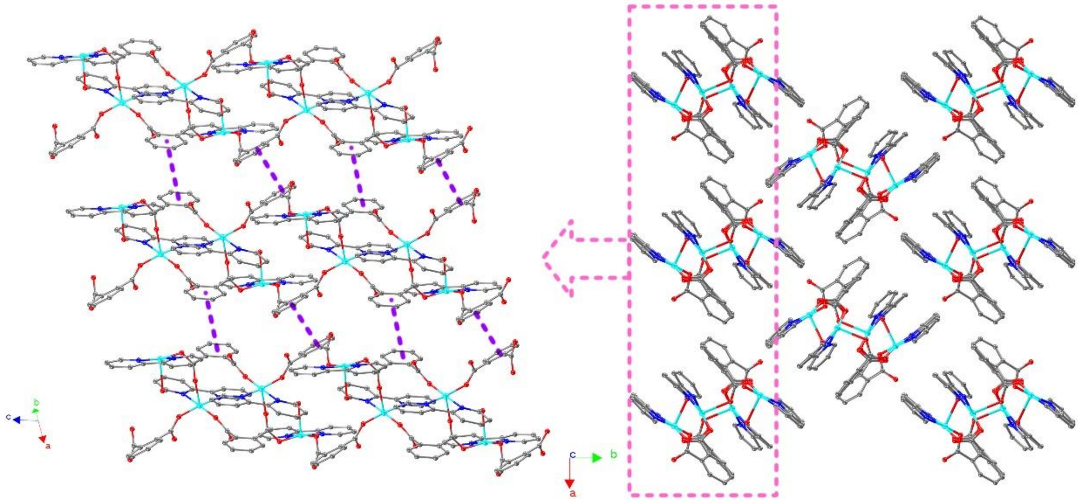

Figure S3. The π-π stacking of μ_2-BDC from adjacent chains (left), and packing diagram of **CP-7** along the *c*-axis (right). Hydrogen atoms bonded to C atoms are omitted for clarity.
